# Chemotherapy of Second Stage Human African Trypanosomiasis: Comparison between the Parenteral Diamidine DB829 and Its Oral Prodrug DB868 in Vervet Monkeys

**DOI:** 10.1371/journal.pntd.0003409

**Published:** 2015-02-05

**Authors:** John K. Thuita, Kristina K. Wolf, Grace A. Murilla, Arlene S. Bridges, David W. Boykin, James N. Mutuku, Qiang Liu, Susan K. Jones, Charles O. Gem, Shelley Ching, Richard R. Tidwell, Michael Z. Wang, Mary F. Paine, Reto Brun

**Affiliations:** 1 Trypanosomiasis Research Centre, Kenya Agricultural Research Institute (TRC-KARI), Kikuyu, Kenya; 2 University of North Carolina Eshelman School of Pharmacy, The University of North Carolina at Chapel Hill, Chapel Hill, North Carolina, United States of America; 3 Department of Pathology and Laboratory Medicine, School of Medicine, The University of North Carolina at Chapel Hill, Chapel Hill, North Carolina, United States of America; 4 Department of Chemistry, Georgia State University, Atlanta, Georgia, United States of America; 5 SVC Associates, Inc., Apex, North Carolina, United States of America; 6 Department of Pharmaceutical Chemistry, The University of Kansas, Lawrence, Kansas, United States of America; 7 Swiss Tropical and Public Health Institute, Basel, Switzerland; 8 University of Basel, Basel, Switzerland; Institute of Tropical Medicine, BELGIUM

## Abstract

Human African trypanosomiasis (HAT, sleeping sickness) ranks among the most neglected tropical diseases based on limited availability of drugs that are safe and efficacious, particularly against the second stage (central nervous system [CNS]) of infection. In response to this largely unmet need for new treatments, the Consortium for Parasitic Drug Development developed novel parenteral diamidines and corresponding oral prodrugs that have shown cure of a murine model of second stage HAT. As a rationale for selection of one of these compounds for further development, the pharmacokinetics and efficacy of intramuscular (IM) active diamidine 2,5-bis(5-amidino-2-pyridyl)furan (DB829; CPD-0802) and oral prodrug2,5-bis[5-(N-methoxyamidino)-2-pyridyl]furan (DB868) were compared in the vervet monkey model of second stage HAT. Treatment was initiated 28 days post-infection of monkeys with T. b. rhodesiense KETRI 2537. Results showed that IM DB829 at 5 mg/kg/day for 5 consecutive days, 5 mg/kg/day every other day for 5 doses, or 2.5 mg/kg/day for 5 consecutive days cured all monkeys (5/5). Oral DB868 was less successful, with no cures (0/2) at 3 mg/kg/day for 10 days and cure rates of 1/4 at 10 mg/kg/day for 10 days and 20 mg/kg/day for 10 days; in total, only 2/10 monkeys were cured with DB868 dose regimens. The geometric mean plasma Cmax of IM DB829 at 5 mg/kg following the last of 5 doses was 25-fold greater than that after 10 daily oral doses of DB868 at 20 mg/kg. These data suggest that the active diamidine DB829, administered IM, should be considered for further development as a potential new treatment for second stage HAT.

## Author Summary

Treatment of human African trypanosomiasis (HAT, sleeping sickness) suffers from a shortage of medicines that are both effective, especially against the second (late) stage of the disease, and safe for patients. The development of new HAT medicines also has been significantly influenced by the perceived need for easily administered oral medicines to reduce the need for hospitalization of patients in resource-poor settings where HAT typically occurs. However, the clinical status of second stage patients is likely to dictate the need for their hospitalization, thus both oral and parenterally administered medicines would be utilised effectively. Therefore, in an effort to develop new medicines that meet efficacy and safety requirements, we evaluated a novel injectable diamidine 2,5-bis(5-amidino-2-pyridyl)furan (DB829; CPD-0802) and its oral prodrug formulation 2,5-bis[5-(*N*-methoxyamidino)-2-pyridyl]furan (DB868) in the vervet monkey model of second stage HAT. Treatment with either compound was initiated 28 days post-infection of monkeys with *T. b. rhodesiense* KETRI 2537. DB829 was dosed at 5 mg/kg/day for 5 consecutive days, 5 mg/kg/day every other day for 5 doses or 2.5 mg/kg/day for 5 consecutive days intramuscularly (IM) while DB868 was administered at 20, 10 or 3 mg/kg/day for 10 consecutive days orally. Clinical and parasitological monitoring was carried out for at least 300 days before the monkeys were declared cured. All IM DB829 and oral DB868 dose regimens were well tolerated. In addition, all monkeys (5/5) treated with IM DB829 were confirmed cured. In contrast, oral DB868 cured only 1/4 monkeys at either 10 or 20 mg/kg and did not cure any monkey when dosed at 3 mg/kg. These results indicate that IM DB829 is a suitable compound for further development as treatment for second stage HAT.

## Introduction

**Fig 1:**
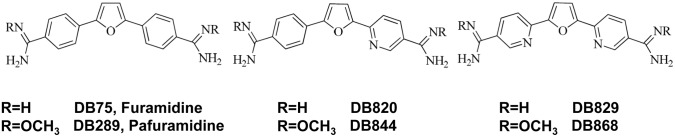
Structures of active diamidines and corresponding prodrugs.

Diamidinesare widely used in the control of both animal and human infectious diseases. Diminazeneaceturate, a diamidine primarily utilized in livestock, has demonstrated activity against *Babesia(bovis* and *bigemina*) and a variety of animal-infective trypanosomes. Pentamidineis an aliphatic diamidine used in humans to treat first (early, haemolymphatic) stage human African trypanosomiasis (HAT), leishmaniasis and *Pneumocystis jirovecii (carinii)* pneumonia [1–3]. Despite the proven clinical utility of pentamidine as a therapeutic agent for first stage *Trypanosomabrucei* (*T. b*.)*gambiense* HAT [3] and the synthesis of potentially more active aromatic diamidines such as furamidine (DB75 (Fig. 1)) [4, 5], no new diamidines have been approved for use against HAT or other human diseases. Since 2000, however, there has been renewed research aimed at developing diamidines with 1) satisfactory bioavailability following oral administration, which would allow for more practical treatment in resource poor settings where HAT is common, and 2) activity against the difficult to treat second stage HAT, which is characterised by trypanosome invasion of the central nervous system (CNS) [6].

To address these objectives, novel diamidineprodrugs were developed by masking the positive charges on diamidines with alkoxy moieties [7]. These prodrugs, especially pafuramidine (DB289 (Fig. 1)), were shown subsequently to have improved absorptive permeability in a human intestinal cell line (Caco-2) compared with the corresponding active diamidine, furamidine (DB75) [8, 9]. Furthermore, the prodrugs pafuramidine and DB844 (Fig. 1) were fully curative in murine models of first and second stage HAT after oral administration [10, 11]. In monkeys, the prodrug pafuramidine was shown to be fully curative against first stage HAT after oral administration [12], providing additional evidence that the prodrug strategy is effective in delivering diamidines to the systemic circulation in different animal species. Despite these successes, cure rates of the prodrugs against second stage HAT in non-human primates ranged from none (0/3) for pafuramidine [12] to a moderate improvement (3/7) for DB844 [13], suggesting the need to identify oral and/or parenterally administered analogues with improved blood-brain barrier (BBB) permeability. Thus, while these prodrugs satisfied the first goal, identifying orally active compounds for the treatment of HAT, they did not satisfy the second goal, identifying compounds with adequate activity against second stage HAT.

The active diamidine 2,5-bis(5-amidino-2-pyridyl)furan (DB829) and its oral prodrug2,5-bis[5-(*N*-methoxyamidino)-2-pyridyl]furan (DB868 (Fig. 1)) were shown to be fully curative in the murine model of second stage HAT (*T. b. brucei* GVR35) [11], suggesting that at least DB829 had acceptable activity in this species. Oral DB868 was subsequently shown to be well-tolerated and fully curative against first stage HAT in monkeys [14]. However, the activity of DB868 and DB829 in a monkey model of second stage HAT remained undetermined. Therefore, the aims of the current study were to 1) investigate the efficacy of both DB829 after intramuscular (IM) administration and DB868 after oral administration in the vervet monkey model of second stage HAT and 2) compare the pharmacokinetics of DB829 and DB868 in infected vervet monkeys.

## Materials and Methods

### Ethics

The experimental guidelines and procedures used in this study were approved (Ref: C/TR/4/325/124) by the Institutional Animal Care and Use Committee at the Trypanosomiasis Research Centre of the Kenya Agricultural Research Institute (TRC-KARI). These IACUC regulations conformed to the “Guidelines for Care and Use of Laboratory Animals in Kenya” provided by the Kenya Veterinary Association.

### Trypanocidal Drugs

DB829, supplied by the Consortium for Parasitic Drug Development (CPDD), was originally synthesised as described [7]. A hydrochloride salt form was synthesised by Scynexis Inc. (Research Triangle Park, NC, USA). The diacetate salt form used in this study (CPD-0802; Lot #SP117-ACE-P5; FW = 451.648) was synthesised by Solvias AG (Basel, Switzerland), by first dissociating the hydrochloride molecule from the Scynexis Inc. compound batch and then recrystalizing in the presence of acetic acid. The final compound had a chemical purity of 97.2% as determined by nuclear magnetic resonance (NMR) and high performance liquid chromatography (HPLC). The compound was protected from light at all times. DB829 was reconstituted in 5% (w/v) dextrose in water to yield 5or 10 mg/mL, and was administered IM to the monkeys at 0.5 mL/kg body weight. Each dose was administered as two aliquots at separate sites on the same limb; subsequent doses were administered on alternating limbs. Dosing solutions were prepared daily immediately before dosing.

DB868, also supplied by the CPDD, was originally synthesised as described [7]. The diacetate salt form used in this study (CPD-007–10; Lot #2-JXS-28; FW = 564.370) was synthesised by Scynexis Inc. and had a chemical purity of 95.6% as determined by NMR and HPLC. As with DB829, DB868 was protected from light at all times. DB868 dosing solutions were prepared daily by reconstituting the drug in distilled de-ionised water to yield1.5, 5, or 10 mg/mL, and were administered orally to the monkeys at 2 mL/kg body weight. Dosing solutions were prepared immediately before dosing.

### Experimental Animals

Eighteen vervet monkeys [*Chlorocebus (Cercopithecus) aethiops*; African green monkeys], weighing between 2.5 and 5.5kg, were used. Following acquisition from the Institute of Primate Research (Nairobi, Kenya), these monkeys were subjected to routine 90-day quarantine procedures designed to ensure a lack of infectious diseases [15, 16]. The monkeys were housed in stainless steel cages and maintained on a diet of fresh fruits and vegetables (bananas, tomatoes, carrots and green maize) and commercial monkey cubes (Unga Feeds, Nakuru, Kenya) fed twice daily [14]. The commercial monkey cubes were manufactured to have the following nutrient composition: crude protein, 19.4% (w/w); crude fiber, 5.6% (w/w); ether extracts that include fats and lipids, 4.2% (w/w); and nitrogen-free extracts, 66.5% (w/w) [14]. Clean drinking water was provided *ad libitum*.

### Study Design

The current study used the KETRI vervet monkey model of HAT, a model developed over 30 years ago through the experimental infection of vervet monkeys with *T. b. rhodesiense* [17]. The resultant infections were found to mimic human sleeping sickness clinically, pathologically, and immunologically [18, 19, 20, 21]. Trypanosomes were identified in peripheral blood first and cerebrospinal fluid (CSF) second, allowing the disease to be classified into an early (haemo-lymphatic) and a late (central nervous system [CNS]) stage, similar to the human disease [18, 19]. This model has been used widely and has been determined to be a reproducible tool for the study of pathogenesis and product (drugs and diagnostics) evaluation [15, 22, 23]. Monkey trypanosomal infections have been determined to be invariably fatal without therapeutic intervention [10], a characteristic also widely accepted as true for human sleeping sickness.

The efficacies of the active drug DB829 (CPD-0802)administered IM and the prodrug DB868 administered orally against the KETRI vervet monkey model of second stage HAT were evaluated in the current study. At the beginning of each experiment, baseline data (weight, clinical and haematological parameters) were collected once every week for 2 weeks, after which monkeys were infected by intravenous injection of approximately 10^4^ *T. b. rhodesiense* KETRI 2537 trypanosomes diluted from the infected blood of immunosuppressed donor Swiss white mice [13–17]. Parasitaemia and CSF parasitosis were monitored using established techniques for blood trypanosomes [24, 25] and CSF trypanosomes [13, 26], respectively. Treatment with DB829 or DB868 was initiated 28 days post-infection (DPI), following confirmation of second stage HAT using the presence of trypanosomes and elevated white cells (> 5 cells/µL) in CSF as biomarkers [3].

Due to a regulated freeze on acquisitions of new non-human primates, only six monkeys were available for the IM DB829 study. These animals were divided into three treatment groups, each consisting of one male and one female, to compare dose-responsiveness between consecutive- and alternate-day dosing. Monkeys were treated with 2.5 or 5 mg/kg/day for 5 consecutive days or with 5 mg/kg/day on alternating days for a total of 5 doses (Table 1).

**Table 1:** Efficacy of intramuscular DB829 against second stage *T. b. rhodesiense* infection in vervet monkeys.

**Parameter/Outcome**	**Intramuscular DB829 (CPD-0802)**						
	**5 mg/kg × 5 days**		**5 mg/kg × 5 alternate days**		**2.5mg/kg × 5 days**		**Overall Results**
**Monkey ID**	**569F**	**659M**	**668M**	**676F**	**546M**	**693F**	
Prepatent period (DPI)	4	4	5	5	4	3	4 (3–5)^a^
Peak parasitaemia (Log_10_ P)	7.2	7.8	7.8	7.5	7.5	8.1	7.7± 0.1^b^
Time to parasitization of CSF (days)	27	27	21	21	27	14	24(14–27)^a^
Peak number of trypanosomes/μL of CSF	1	1	1	1	1	1	1
Trypanosomes at EoT	Neg	Neg	Neg	Neg	Neg	Neg	6/6
Provisional efficacy at 100 days post-LDD of DB829	Cured	Cured	Cured	Cured	Cured	Cured	6/6
Final efficacy assessment at 300 days post-LDD of DB829	Cured	WD (91)	Cured	Cured	Cured	Cured	5/5
Cured/treated	1/1		2/2		2/2		

Key: DPI, days post-infection; P, parasitaemia; CSF, cerebrospinal fluid; EoT, end of treatment; LDD, last drug dose; F, female; M, male; Neg, Negative; WD (91), withdrawn 91 days post-LDD; a, median (range); b, mean ± standard error of mean.

Twelve additional monkeys were available for the oral DB868 efficacy study. These animals were, before infection, divided into three groups, each consisting of two males and two females. DB868 was administered orally to each group at 3, 10 or 20 mg/kg/day for 10 consecutive days (Table 2). However, two monkeys that had been allocated to the 3 mg/kg/day dose-group did not become blood or CSF trypanosome positive and were consequently withdrawn from the study, leaving only two monkeys, a male (monkey 687) and a female (monkey 670), in that group (Table 2).

**Table 2:** Efficacy of oral prodrug DB868 against second stage *T. b. rhodesiense* infection in vervet monkeys.

**Parameter/Outcome**	**Oral DB868**										
	**20 mg/kg × 10 days**				**10 mg/kg × 10 days**				**3 mg/kg × 10 days**		**Overall Results**
**Monkey ID**	**573F**	**679F**	**689M**	**696M**	**688M**	**690F**	**695M**	**697F**	**670F**	**687M**	
Pre-patent period (DPI)	4	5	5	4	5	5	4	4	7	4	4.5 (3–7)^a^
Peak parasitaemia (Log_10_ P)	8.1	7.8	7.8	7.8	7.5	7.8	7.5	7.8	7.2	7.8	7.8 ± 0.1^b^
Time to parasitization of CSF (days)	14	27	27	7	21	14	7	7		21	10.5 (7–27)^a^
Peak number of trypanosomes/μL of CSF	2	1	1	1	1	1	1	1		2	1 (1–2)^a^
Provisional efficacy at EoT	Cured	Cured	Cured	Cured	Cured	Cured	Cured	Cured	Not cured	Not cured	8/10
Provisional efficacy at 100 days post-LDD of DB868	R	Cured	Cured	Cured	R	Cured	R	R			4/10
Final efficacy assessment at 300 days post-LDD of DB868	R	Cured	R	R	R	Cured	R	R			2/10
Cured/treated	1/4				1/4				0/2		

Key: DPI, days post-infection; P, parasitaemia; CSF, cerebrospinal fluid; EoT, end of treatment; LDD, last drug dose; F, female; M, male; R, relapsed; a, median (range); b, mean ± standard error of mean.

Parasitaemia post-infection was monitored daily, using ear-prick capillary blood, through the completion of DB868 and DB829 treatment. Following the last drug dose (LDD), blood and CSF samples were examined for trypanosomes at 1, 4, 7, and 14 days to determine the end of treatment (EoT) response; monkeys which were positive for trypanosomes in either blood or CSF at the end of this 14-day period were classified as treatment failures/not cured while those that were free of trypanosomes were classified as provisionally cured. After the 14-day EoT evaluations, parasitaemia examinations were continued twice weekly to ≥ 300 days post-LDD as described previously [12, 13]. Additionally, physical examinations and haematological assessments were carried out once weekly up to 28 days post-LDD, once every two weeks between 28 and 100 days post-LDD, and monthly thereafter until the end of the study as described previously [12, 13]. At 300 days post-LDD, monkeys were considered cured if they remained normal clinically and parasite-free in the blood and CSF. Pharmacokinetic outcomes were monitored for a minimum of 60 days post-LDD as described previously [13]. To facilitate the collection of blood and CSF for these assessments, the monkeys were anaesthetised with an IM injection of ketamine HCl (10–15 mg/kg) and diazepam (0.5 mg/kg). Monkeys that relapsed, characterized by trypanosome recrudescence in the blood or CSF, were rescue-treated with melarsoprol. Melarsoprol (Mel B), supplied by the World Health Organization as a 3.6% (w/v) solution in propylene glycol, was administered intravenously at 3.6 mg/kg/day for 4 consecutive days.

### HPLC-MS/MS Quantification of DB868 and DB829 in Monkey Plasma

Plasma samples were processed for quantification of DB868 and DB829 by high performance liquid chromatography-tandem mass spectrometry (HPLC-MS/MS) as described previously [14]. CSF samples were processed in a manner similar to that of plasma samples.

### Criteria for Euthanasia of Monkeys

The study monkeys that relapsed after treatment were withdrawn from the study and humanely euthanized. The humane endpoints for these animals were determined as described in “recognition and alleviation of pain in laboratory animals” (http://www.nap.edu/catalog/12526.html). The key clinical parameters that were monitored and used to aid decision making on when to euthanize study subjects included a monkey’s inability or reluctance to perch and consumption of less than 1/4 of normal daily feed intake for 2–3 consecutive days as summarized previously [13, 14, 16]. The affected monkeys were euthanized by intravenous administration of 20% (w/v) pentobarbitone sodium solution (150 mg/kg body weight).

### Data Analysis

Clinical and haematological data were analysed using Statview for Windows Version 5.0.1 (1995–1998; SAS Institute Inc., Cary, NC, USA). Repeated measures ANOVA, with Fisher’s PLSD post hoc test, was used to test the effect of trypanosome infection on haematologic parameters in comparison with respective baseline values (α = 0.05). In addition, the effects of IM DB829 and oral DB868 on these haematologic parameters in monkeys with confirmed second stage HAT were tested. Pharmacokinetic outcomes were recovered using traditional non-compartmental methods using Phoenix WinNonlin (version 6.2; Pharsight, Mountain View, CA, USA).

## Results

### Parasitaemia, CSF Parasitosis, and Clinical Disease Prior to Treatment

**Fig 2:**
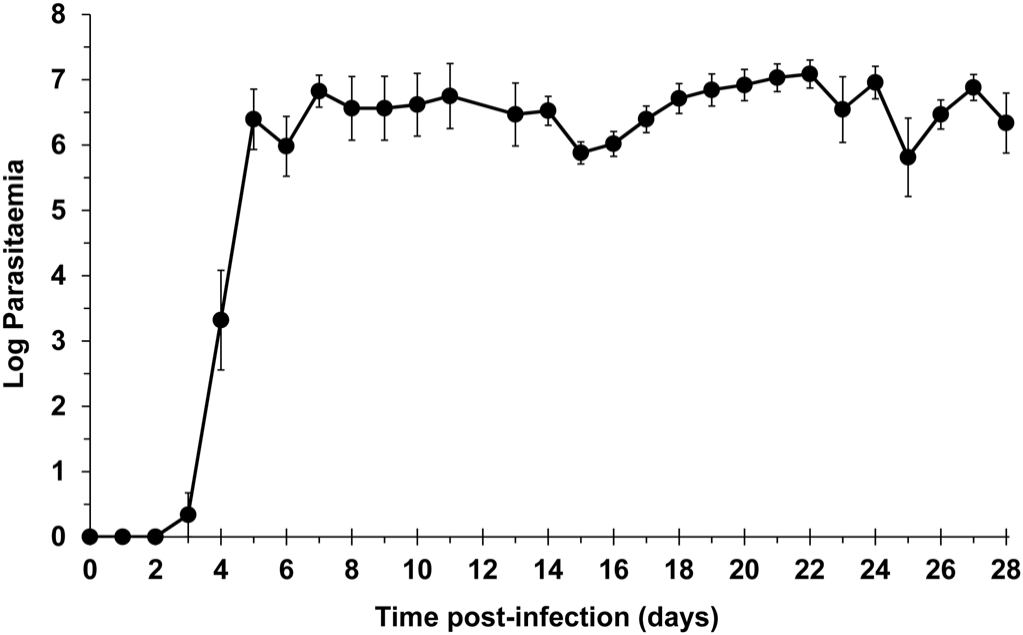
Parasitaemia progression in vervet monkeys following infection. Monkeys were infected with *T. b. rhodesiense* KETRI 2537 on Day 0. Symbols represent means and standard error of the mean (n = 16).

Following intravenous injection of trypanosomes, median (range) pre-patent periods of 4 (3–5) and 4.5 (4–7) days were observed in the DB829and DB868 efficacy studies, respectively (Tables 1 and 2). Peak parasitaemia reached 10^7^ trypanosomes/mL of blood in individual monkeys (Tables 1 and 2), with fluctuations characteristic of trypanosomal infection as demonstrated by the change in mean parasitaemia (Fig. 2); these observations are similar to primary parasitaemia data previously reported for this model [13]. Clinically, first stage disease was evident by transient inappetance, rough haircoats, fluctuating fever, and weight loss (< 5%). General lymphadenopathy and enlargement of the spleen (up to 3-times the pre-infection size) also were observed.

**Fig 3:**
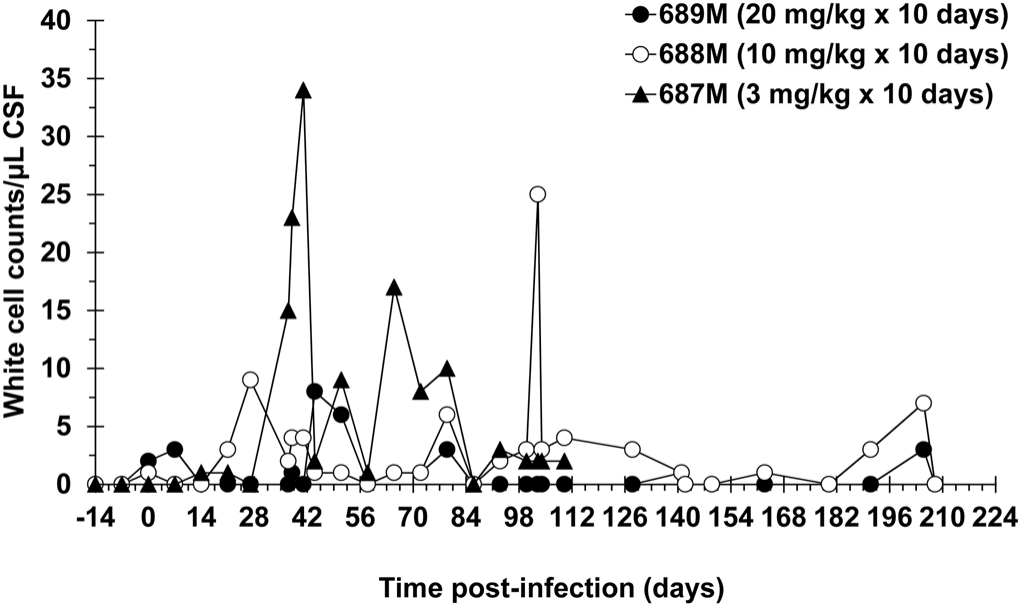
White cell changes in the cerebrospinal fluid of infected monkeys treated with the diamidine prodrug DB868. DB868 was administered orally at 20, 10, or 3 mg/kg/day for 10 days 28–37 days post-infection with *T. b. rhodesiense* KETRI 2537. M, male.

CSF parasitosis was confirmed in the experimental monkeys at or before the last pre-treatment sampling point at 27 DPI, at a density of 1–2 trypanosomes/μL of CSF (Tables 1 and 2), confirming that these monkeys had progressed to second stage disease [3]. However, monkey 670F, which did not have detectable trypanosomes in CSF, had elevated CSF white cell numbers compared with baseline and was therefore included in the study (Table 2). Two others, monkeys 646M and 675F, which also were inoculated with trypanosomes with the objective of treating them with oral DB868 at 3 mg/kg/day for 10 days, were withdrawn from the study because they did not become trypanosome positive in blood or CSF and also did not have pathological numbers of CSF white cells. The failure of these two monkeys to become infected may be due to their participation in a diamidine toxicity study 7 months prior to the start of the current study. The response of CSF white cells to infection in the current groups of infected monkeys exhibited wide variations between individuals, as shown for three monkeys that were treated with DB868 (Fig. 3). Clinically, a limited number of monkeys (monkeys 668M, 688M, 689M, and 693F) exhibited signs suggestive of second stage disease, including altered behaviour and chirping. The monkeys were treated starting at 28 DPI with different dose regimens of eitherDB829 or DB868 (Tables 1 and 2).

### Efficacy of DB829 Dose Regimens

**Fig 4:**
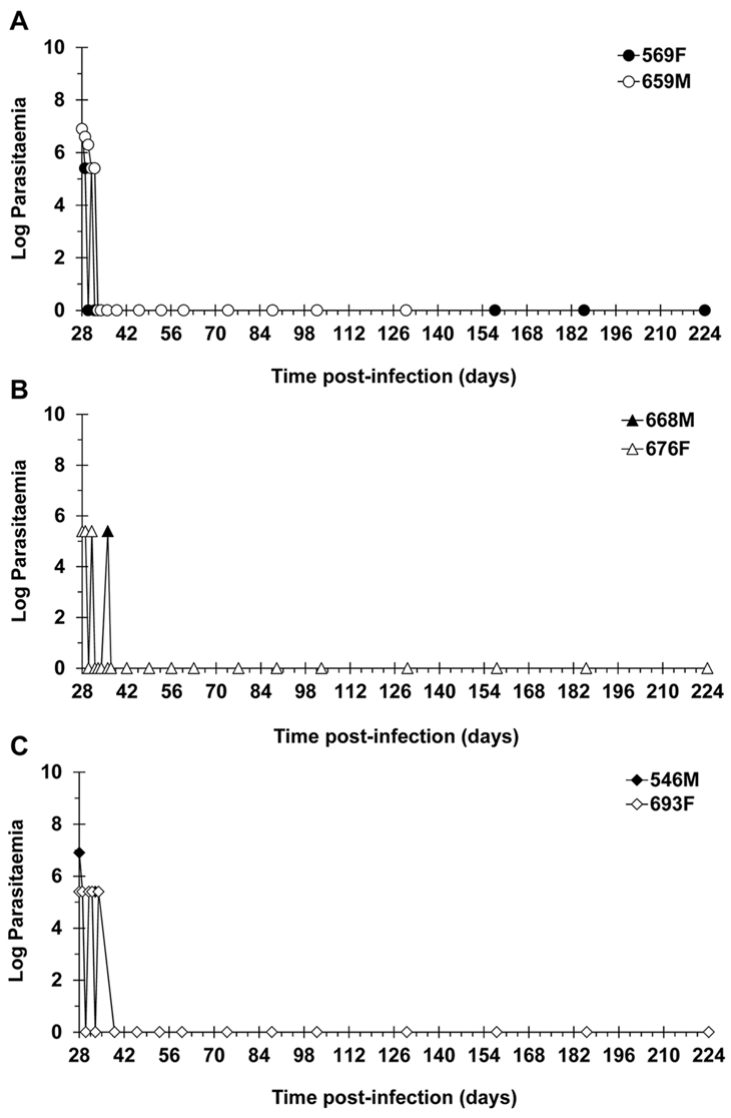
Resolution of parasitaemia in infected vervet monkeys treated with the active diamidine DB829. Monkeys were infected with *T. b. rhodesiense* KETRI 2537 and later confirmed to have progressed to second stage HAT, were administered with DB829 intramuscularly at (**A**) 5 mg/kg/day for 5 consecutive days (28–32 days post-infection), (**B**) 5 mg/kg/day for 5 alternate days (28, 30, 32, 34 and 36 days post-infection), or (**C**) 2.5 mg/kg/day for 5 consecutive days (28–32 days post-infection). F, female; M, male.

DB829, the active diamidine, was administered IM at 5 mg/kg/day for 5 consecutive days (monkeys 569F and 659M), 5 mg/kg/day every alternate day for 5 doses (monkeys 668M and 676F), or 2.5 mg/kg/day for 5 consecutive days (monkeys 546M and 693F; Table 1). Monkeys that were treated with either 5 mg/kg/day dose regimen were blood and CSF parasite negative 1 day post-LDD (Table 3). In contrast, trypanosomes were more persistent in monkeys dosed with DB829 at 2.5 mg/kg/day; these monkeys were first detected to be negative for trypanosomes in both blood and CSF at 4 days (monkey 693F) and 7 days (monkey 546M) post-LDD (Table 3). Subsequently, trypanosomes were undetectable in the blood or CSF of these two monkeys throughout the remaining EoT evaluation period, contributing to a combined provisional cure rate by DB829 of 6/6. Five out of the six monkeys (monkeys 569F, 668M, 676F, 546M and 693F) remained parasite-free in blood and CSF for more than 300 days post-LDD (Table 1; Fig. 4) and were declared cured. Monkey 659M (5 mg/kg/day for 5consecutive days) developed pneumonia and had to be euthanized 91 days post-LDD. Trypanosome recrudescence was not observed in blood or CSF collected from this monkey at any time point post-treatment. However, since the mandatory post-treatment monitoring period of 300 days was not completed, the monkey could not be classified as cured and was considered withdrawn from the study (Table 1). Overall, the final combined cure rate with DB829 treatments was 5/5 (Table 1).

**Table 3:** Time to clearance of trypanosomes from body fluids of monkeys treated with DB829 or DB868.

**Monkey ID**	**Drug (Route)**	**Dose regimen**	**Time to initial clearance of trypanosomes from body fluids (blood and CSF)** **(days post-LDD)**	**Time to relapse (days post-LDD)**
569F	DB829 (IM)	5mg/kg × 5 days, consecutive-day dosing 28–32 DPI	1	N/A
659M			1	N/A
668M	DB829 (IM)	5 mg/kg × 5 days, alternate-day dosing (28, 30, 30, 32, 34 and 36 DPI)	1	N/A
676F			1	N/A
546M	DB829 (IM)	5mg/kg × 5 days, consecutive-day dosing 28–32 DPI	4	N/A
693F			7	N/A
573F	DB868 (Oral)	20 mg/kg × 10 days, 28–37 DPI	7	77
679F			1	N/A
689M			1	161
696M			4	133
688M	DB868 (Oral)	10 mg/kg × 10 days, 28–37 DPI	1	56
690F			1	N/A
695M			1	28
697F			1	28
670F	DB868 (Oral)	3 mg/kg × 10 days, 28–37 DPI	N/A	N/A
687M			7	14

Key: F, female; M, male; IM, intramuscular; DPI, days post-infection; CSF, cerebrospinal fluid; LDD, last drug dose;

N/A, not applicable (monkey did not relapse)

### Efficacy of DB868 Dose Regimens

**Fig 5:**
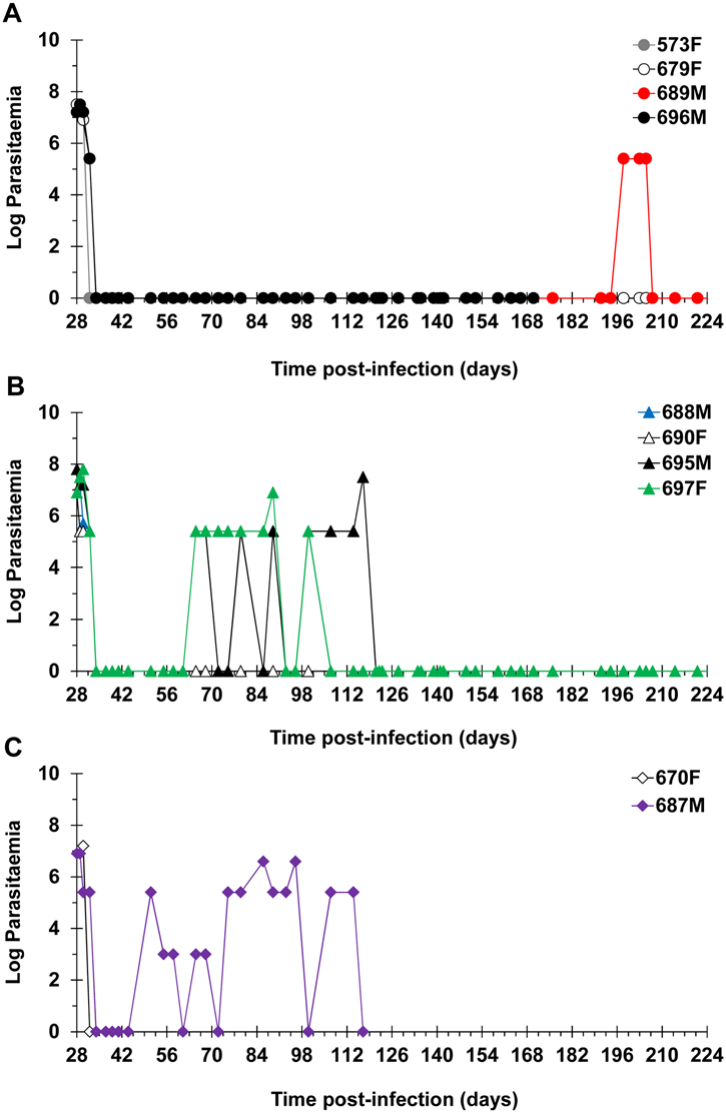
Primary and relapse parasitaemia in infected vervet monkeys treated with the diamidine prodrug DB868. DB868 was administered orally at (**A**) 20 mg/kg/day, (**B**) 10 mg/kg/day, or (**C**) 3 mg/kg/day for 10 consecutive days following infection with *T. b. rhodesiense* KETRI 2537 (28–37 days post-infection); the monkeys were confirmed to have progressed to second stage HAT before initiation of treatment. Relapse infections occurred at different times in specific individuals. Relapsed monkeys were rescue-treated with melarsoprol administered intravenously at 3.6 mg/kg/day for 4 consecutive days. F, female; M, male.

The prodrug DB868 was administered orally to groups of monkeys at 20, 10 and 3mg/kg/day for 10 consecutive days. The four monkeys dosed with DB868 at 20 mg/kg/day became aparasitaemic by the 7^th^ day of treatment at 34 DPI (Fig. 5). CSF trypanosomes were persistent in some monkeys (monkeys 573F and 696M) but all were cleared 7 days after the LDD (Table 3), allowing all monkeys to be classified as provisionally cured upon completion of the 14-day EoT evaluation period (Table 2). However, 3/4 monkeys (monkeys 573F, 689M and 696M)relapsed, resulting in a final cure rate of 1/4 after the minimum 300 days of post-treatment monitoring (Table 2). The median (range) time to relapse was 133 (77–161) days post-LDD (Table 3). The three relapsed monkeys were confirmed to have CSF trypanosomes, CSF white cell aberrations (Fig. 3) and/or clinical signs of CNS disease; however, only one (monkey 689M) was diagnosed to have trypanosomes in the blood (Fig. 5). Clinically, monkey 696M deteriorated quickly, necessitating euthanasia 130 days post-LDD of DB868. The remaining two relapsed animals (monkeys 573F and 689M) were successfully rescue-treated with melarsoprol starting at 81 and 168 days post-LDD of DB868, respectively.

All four monkeys that were treated with oral DB868 at 10 mg/kg/day for 10 days responded in a comparable manner to the higher (20 mg/kg/day) dose group; all became blood parasite negative by the 7^th^ day of dosing at 34 DPI (Fig. 5) and were negative for trypanosomes in both blood and CSF at the EoT evaluation (Table 2). In addition, 3/4 monkeys (monkeys 688M, 695M and 697F) relapsed, resulting in a final cure rate of 1/4 (Table 2). However, the median (range) time to relapse, 28 (28–56) days post-LDD of DB868, was shorter than that of the higher dose group (Table 3). All three relapsed monkeys (monkeys 688M, 695M and 697F) were positive for blood trypanosomes (Fig. 5), while only two (monkeys 688M and 697F) had clinical signs and/or clinico-pathological indicators of CNS disease (Fig. 3). Clinical signs of CNS involvement in the two monkeys included ataxia, circling and altered behaviour. The three relapsed monkeys were successfully rescue-treated with melarsoprol starting at either 63 days (monkeys 688M and 697F) or 81 days (monkey 695M) post-LDD of DB868 (100 or 118 DPI, respectively).

The two monkeys treated with DB868 at 3 mg/kg/day for 10 days (monkeys 670F and 687M) (Table 2) also experienced a brief period of aparasitaemia, which started immediately after the 7^th^ drug dose at 34 DPI (Fig. 5). However, the clinical condition of monkey 670F did not improve post therapy, necessitating euthanasia 1 day post-LDD of DB868. In monkey 687M, blood trypanosomes reappeared 14 days post-LDD (51 DPI) (Fig. 5; Table 3) while CSF trypanosomes were not eliminated; taken together, these data indicated that the 3 mg/kg/day dose regimen did not achieve cure in any monkey (Table 2). The relapse parasitaemia in monkey 687M remained near the limit of detection (antilog 5.4 [24]) and tended to be lower in intensity compared to the original parasitaemia in the same monkey (Fig. 5). In contrast, CSF white cell aberrations were more pronounced in relapse than the period prior to treatment (Fig. 3). The monkey was rescue-treated with melarsoprol starting at 81 days post-LDD of DB868 (118 DPI). However, only 2/4 daily doses of melarsoprol were administered, after which the monkey’s clinical condition deteriorated significantly, necessitating euthanasia.

In summary, a combined cure rate of 2/10 was achieved with oral DB868. Two monkeys were not provisionally cured and six monkeys eventually relapsed. Of those that relapsed, 5/6 were successfully rescue-treated with melarsoprol; the final monkey had to be euthanized prior to the start of rescue-treatment.

### Infection- and Treatment-Induced Haematological Changes

**Fig 6:**
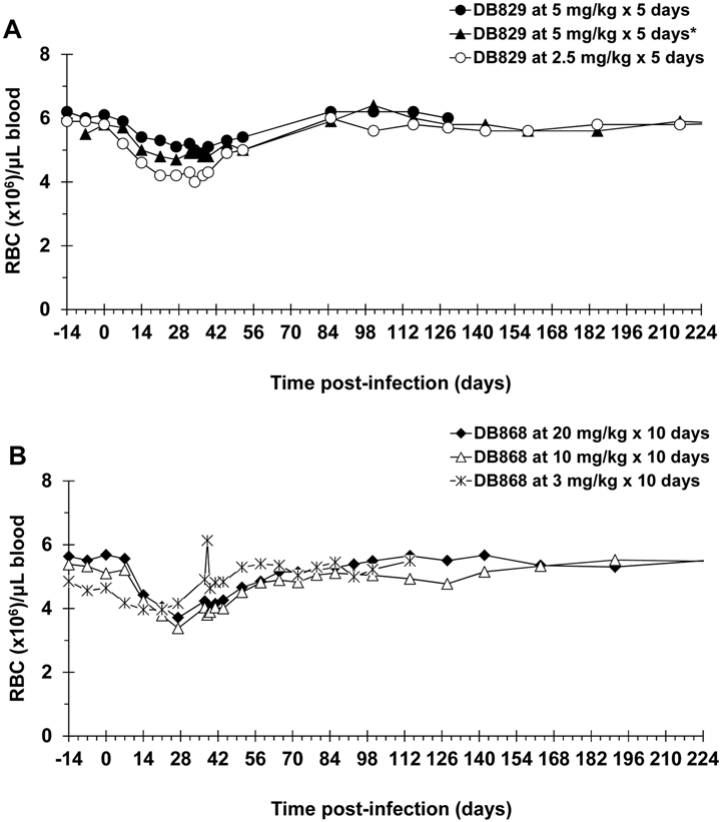
Red blood cell density changes in infected vervet monkeys treated with experimental drugs. Monkeys were infected with *T. b. rhodesiense* KETRI 2537 and upon confirmation of onset of second stage HAT, treated with (**A**) DB829 or (**B**) DB868. DB829 was administered intramuscularly for either 5 consecutive days (28–32 days post-infection) or 5 alternate (*) days (28, 30, 32, 34 and 36 days post-infection). DB868 was administered orally for 10 consecutive days (28–37 days post-infection). Symbols represent means (**A**; n = 2) or means and standard error of the mean (**B**; n = 4).

In all DB829 and DB868 treatment groups, red blood cell (RBC) counts exhibited an infection-induced decline that was most prominent at the last pre-treatment sampling point, 27 DPI (Fig. 6). For the monkeys in the DB829 study (n = 6), the decline in RBC counts was significant (*p* < 0.0001), from a baseline (0 DPI) mean (± SE) of 5.9 ± 0.3 to 4.7 ± 0.3 at 27 DPI. These monkeys also had a significant decline in haematocrit (*p* < 0.0001), from a mean (± SE) of 48.8 (± 2.6)% at baseline (0 DPI) to 35.5 (± 1.8)% at 27 DPI, a 27.3% decrease. Similarly, monkeys in the DB868 study (n = 10) experienced significant infection-related declines (*p* < 0.0001) in both RBC counts and haematocrit. The mean corpuscular volume and mean corpuscular haemoglobin for all groups of monkeys also declined significantly (*p* < 0.05), consistent with previous findings in monkeys infected with the same *T. b. rhodesiense* KETRI 2537 isolate [13]. Upon treatment with DB829 IM or DB868 orally, RBC counts recovered to baseline levels within approximately 1–2 months (Fig. 6). The average RBC counts of all treatment groups remained stable throughout the remainder of the study (Fig. 6), despite the occurrence of relapse at various times for different monkeys treated with DB868.

**Fig 7:**
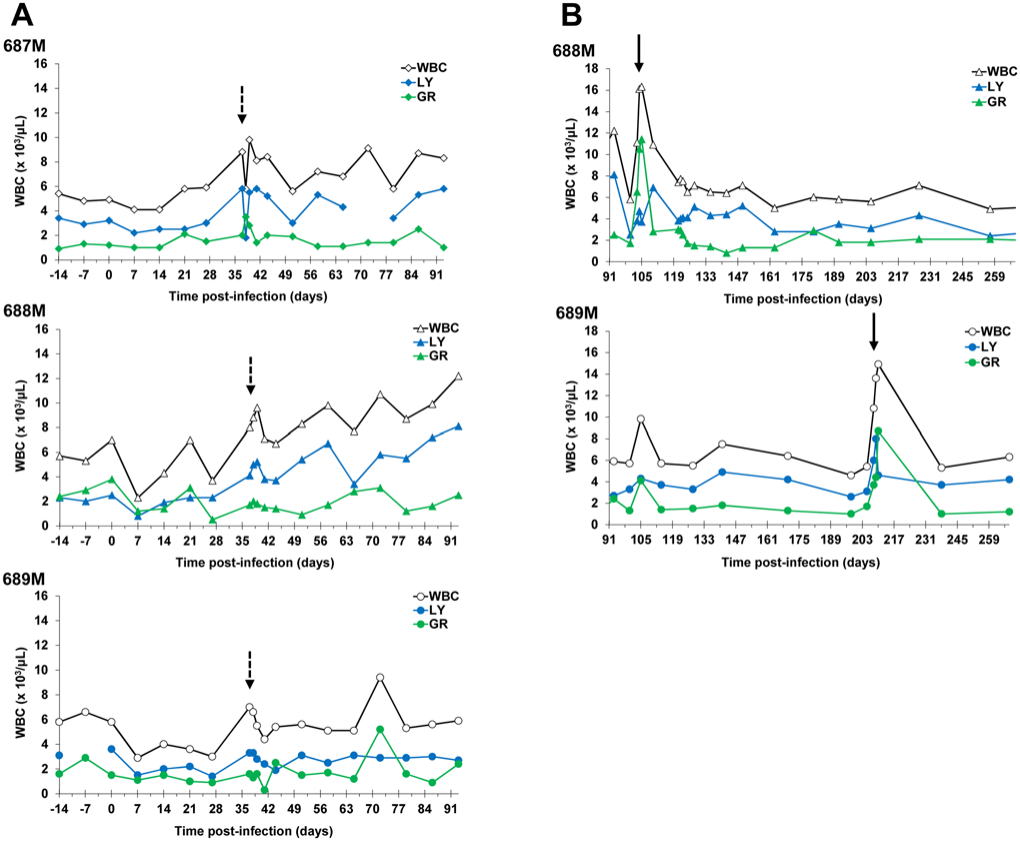
White cell changes in the blood of three infected monkeys treated with the diamidine prodrug DB868. DB868 was administered orally at 20 mg/kg/day (689M), 10 mg/kg/day (688M), or 3 mg/kg/day (687M) for 10 days after infection with *T. b. rhodesiense* KETRI 2537 (28–37 days post-infection). Confirmed relapses were rescue-treated with melarsoprol intravenously at 3.6 mg/kg/day for 4 consecutive days. White blood cell changes from (**A**) -14 to 93 days and (**B**) 93 to 268 days post-infection. M, male; WBC, white blood cells; LY, lymphocytes; GR, granulocytes. Dashed arrows, last DB868 dose; bold arrows, last melarsoprol dose.

Infection-related thrombocytopenia was observed between 7–27 DPI (Table 4). Within one week post-LDD of DB868, platelet cell counts had recovered to baseline (pre-infection) levels. Similarly, leukopenia was observed at 7 DPI (Table 4) and was largely due to declines in lymphocyte and granulocyte numbers (Fig. 7A); monocyte numbers remained stable throughout. However, unlike RBC and platelet counts, which remained low prior to therapeutic intervention with DB829 or DB868, white blood cell (WBC) densities exhibited wide variability between individuals, rebounding in some monkeys while remaining low in others (Table 4; Fig. 7A). Upon treatment with DB829 or DB868, mean WBC counts for all treatment groups returned to baseline levels (Table 4). In individual monkeys, however, WBC counts continued to fluctuate, especially in those monkeys that eventually relapsed (Fig. 7A and 7B). The highest WBC peaks that occurred in the relapsed monkeys were observed approximately 1–4 days post-LDD of melarsoprol (Fig. 7B). These peaks were largely the result of lymphocyte and granulocyte increases (Fig. 7B).

**Table 4:** Changes in blood platelet and white cell counts following infection of monkeys with *T. b. rhodesiense* and subsequent treatment within tramuscular DB829 and oral DB868.

**Drug**	**Dose (mg/kg × d)**	**DPI (DPT)**	**0**	**7**	**27**	**38 (1)**	**44 (7)**	**65 (28)**	**100 (63)**	**137 (100)**	**224 (180)**
DB829	5 × 5^a^	PLT	424 ± 60	112 ± 11	156 ± 42	464 ± 32	515 ± 39	419 ± 4.5	352 ± 44	367 ± 60	N/A
		WBC	6.0 ± 0.4	3.4 ± 0.5	4.6 ± 0.2	7.8 ± 0.3	6.6 ± 0.5	7.3 ± 1.0	5.3 ± 1.1	4.5 ± 0.1	N/A
	5 × 5^b^	PLT	312 ± 31	96 ± 12	200 ± 2	328 ± 7.5	395 ± 26	378 ± 71	246 ± 26	327±25	250 ± 53
		WBC	4.8 ± 0.5	3.3 ± 1.1	4.7 ± 0.6	4.8 ± 0.6	5.6 ± 0.5	6.5 ± 0.7	5.8 ± 0.8	6.3 ± 0.3	6.0 ± 0.3
	2.5 × 5^a^	PLT	285 ± 23	54 ± 20	115 ± 32	265 ± 17	332 ± 27	292 ± 5	242 ± 64	327 ± 27	389 ± 13
		WBC	5.8 ± 2.1	3.9 ± 1.4	5.4 ± 1.3	6.8 ± 1.5	8.9 ± 3.4	8.1 ± 2.1	6.3 ± 1.0	6.0 ± 1.6	6.7 ± 1.9
DB868	20 × 10^a^	PLT	323 ± 42	112 ± 15^S^	131 ± 32^S^	410 ±14	362±57	334±52	318± 31	285± 51	243±50
		WBC	8.1± 1.5	3.5 ± 0.8^S^	4.3 ± 0.7^S^	7.4 ± 0.8	6.5± 0.6	5.9 ± 0.3^S^	6.9 ± 0.6	8.1 ± 0.6	9.7± 2.9
	10 × 10^a^	PLT	273 ± 76	75 ± 13	54 ± 4	292 ± 41	331 ± 90	307 ± 82	217 ± 32	451 ± 65	337 ± 19
		WBC	5.7 ± 0.3	2.7 ± 0.2^S^	4.3 ± 0.6	8.9 ± 0.3	7.1 ± 0.8	6.9 ± 0.7	7.9 ± 1.7	6.2 ± 0.5	6.4 ± 0.6
	3 × 10^a^	PLT	355 ± 215	259 ± 186	126 ±33	472 ±248	N/A				
		WBC	5.7 ± 0.8	4.7 ± 0.6	6.4 ± 0.5	8.2 ± 0.6	N/A				

Key: d, day;DPI, days post-infection; DPT, days post-treatment (post-last drug dose of DB829 or DB868); a, consecutive-day dosing; b, alternate-day dosing; PLT, platelet counts;WBC, white blood cell counts; N/A, not applicable; S, *p* < 0.05 (repeated measures ANOVA with Fisher’s PLSD post hoc test).

### Pharmacokinetics

**Fig 8:**
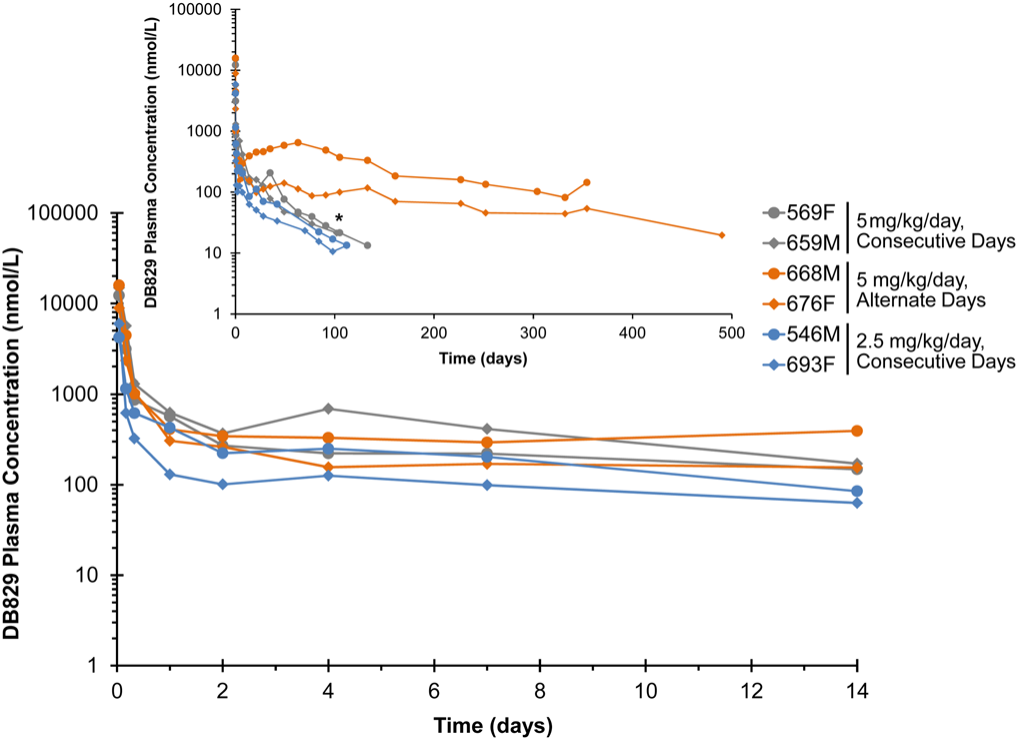
Plasma concentration-time profiles following administration of the active diamidine DB829 to infected vervet monkeys. Monkeys confirmed to have second stage HAT were administered DB829 intramuscularly, beginning at 28 days post-infection with *T. b. rhodesiense* KETRI 2537, at 5 mg/kg/day for 5 consecutive days, 5 mg/kg/day for 5 alternate days or 2.5 mg/kg/day for 5 consecutive days. The inset graph shows the extended profile out to the end of the study. * denotes the time (91 days post-last drug dose) that monkey 659 was euthanized due to non-drug related complications (pneumonia). F, female; M, male.

Following IM injection of the active diamidine DB829 (Fig. 8), the median plasma T_max_ of the 2.5and 5 mg/kg/day consecutive-day dose groups were similar (0.04 days or 1 h; Table 5). The geometric mean C_max_ and AUC for the 5 mg/kg/day group was 2.7-and 2.1-fold higher, respectively, than that for the 2.5 mg/kg/day dose group (Table 5). The geometric mean (range) terminal half-life (t_½_) for the consecutive-day dosing regimens was 38 (33–44) days. Geometric mean C_max_ and median T_max_ for alternate-day dosing with 5 mg/kg/day were comparable to those for consecutive-day dosing (Table 5). The AUC and the terminal half-life for the alternate-day dosing regimen were 5- and 4-fold greater, respectively, than corresponding values for the consecutive-day dosing regimen (Table 5).

**Table 5:** Pharmacokinetics of DB829 in vervet monkeys with second stage HAT after the 5^th^ intramuscular dose.

		**Group 1**		**Group 2**		**Group 3**	
**Outcome**	**Units**	**569F**	**659M**	**668M**	**676F**	**546M**	**693F**
T_max_	day	0.04	0.04	0.04	0.04	0.04	0.04
C_max_	nmol/L	12000	15000	16000	8900	4200	5900
AUC_last_ ^†^	nmol/L•day	12000	13000	97000	35000	7700	4500
AUC_0-∞_	nmol/L•day	13000	15000	120000	40000	8300	5300
Cl/F	L/day/kg	0.85	0.76	0.09	0.28	0.67	1.0
t_½_	day	34	44	130	180	33	42

Group 1 = 5 mg/kg × 5 consecutive days; Group 2 = 5 mg/kg × 5 alternating days; Group 3 = 2.5 mg/kg × 5 consecutive days; F, female; M, male; T_max_, time to reach C_max_; C_max_, maximum concentration; AUC_last_, AUC from time 0 to the last measurable concentration; AUC_(0-∞)_, AUC from 0 to infinite time; Cl/F, apparent clearance; t_½_, terminal half-life;

^†^ = last measurable concentration varied between monkeys (112–490 days).

**Fig 9:**
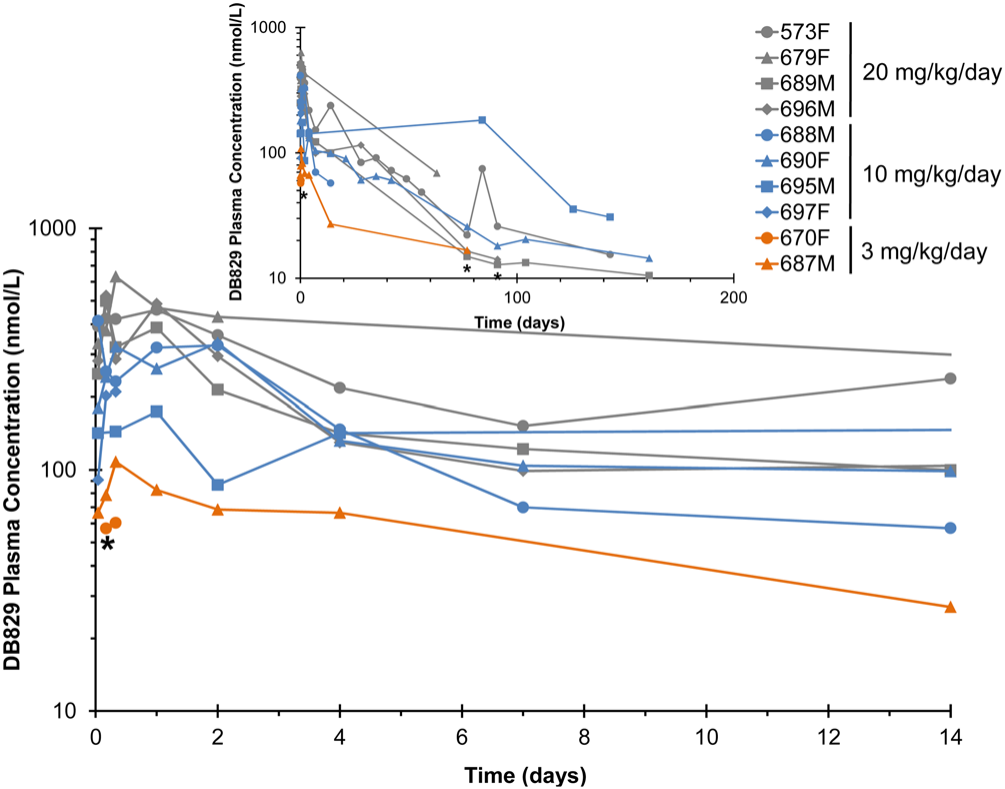
Plasma concentration-time profiles of DB829 following administration of the diamidine prodrug DB868 to infected vervet monkeys. Monkeys confirmed to have second stage HAT were administered DB868 orally, beginning at 28 days post-infection with *T. b. rhodesiense* KETRI 2537, at 20, 10 or 3 mg/kg/day for 10 consecutive days. The inset graph shows the extended profile out to the end of the study. * denotes the time that monkey 670 (1 day post-last drug dose), monkey 687 (82 days post-last drug dose) and monkey 696 (130 days post-last drug dose) were euthanized due to clinical morbidity. F, female; M, male.

Following oral administration of DB868, the prodrug was detected within 1 h post-LDD in all monkeys, with geometric mean plasma concentrations (range) of 120 (100–150) nmol/L for the 3 mg/kg/day group, 750 (420–1500) nmol/L for the 10 mg/kg/day group, and 810 (370–1300) nmol/L for the 20 mg/kg/day group. Concentrations declined rapidly and were undetectable after 8 h in all monkeys, precluding accurate recovery of pharmacokinetic outcomes for DB868. Oral DB868 was converted to the active drug DB829 in all the monkeys (Fig. 9). Median plasma T_max_ for DB829 was 0.33 days (8 h) for the three treatment groups, ranging from 0.04–2.0 days (1–48 h; Table 6). Geometric mean C_max_ (range) was 85 (60–110) nmol/L for the 3 mg/kg/day group, 270 (180–410) nmol/L for the 10 mg/kg/day group, and 530 (460–630) nmol/L for the 20 mg/kg/day group (Table 6). Because rescue-treatment with melarsoprol started as early as 63 days post-LDD of DB868 due to relapse (monkeys 688M and 697F), only the partial exposure (AUC_0–63_) of DB829 was evaluated for all DB868-treated monkeys. The geometric mean AUC_0–63_ for the 10 mg/kg/day and 20 mg/kg/day groups were approximately 3- and 4-fold greater, respectively, than that recovered for the single monkey in the 3 mg/kg/day group (Table 6). An accurate terminal elimination half-life was not recoverable for all monkeys (Table 6), precluding dose-comparisons.

**Table 6:** Pharmacokinetics of DB829 Pharmacokinetics of DB829 in vervet monkeys with second stage HAT after the 10^th^ oral dose of DB868.

	**Monkey**	**T_max_ (day)**	**C_max_ (nmol/L)**	**AUC_0–63_ (nmol/L•day)**	**t_½_ (day)**
20 mg/kg × 10 days	573F	1	460	7700	48
	679F	0.33	630	13000	NC^‡^
	689M	0.17	500	4700	180
	696M	0.17	530	5900	NC^‡^
10 mg/kg × 10 days	688M	0.04	410	2500	NC^‡^
	690F	2.0	340	5100	120
	695M	NC*	NC*	NC*	NC*
	697F	0.33	210	NC^†^	NC^†^
3 mg/kg × 10 days	670F	0.33	60	NC^†^	NC^†^
	687M	0.33	110	1800	NC^‡^

T_max_, time to reach C_max_; C_max_, maximum concentration; AUC_0–63_, AUC from 0 to 63 days; t_½_, terminal half-life; F, female; M, male; NC, not calculable;

* = data was too sparse to determine the T_max_, C_max_, AUC_0–63_, and t_½_ with confidence;

^†^ = data was too sparse to determine the AUC_0–63_, and t_½_ with confidence;

^‡^ = data was insufficient in the terminal phase to determine the t_½_ with confidence.

CSF active drug (DB829) concentrations were determined only for monkeys treated with DB829IM; CSF concentrations were not quantifiable in monkeys treated with oral DB868. Concentrations in the 2.5 mg/kg/day consecutive-day dose group were below the limit of quantification (5 nmol/L). In the 5 mg/kg/day consecutive-day dose group, the geometric mean DB829 concentration was 14 nmol/L at 1 h post-LDD; concentrations decreased to below the limit of quantification by 28 days post-LDD. In the 5 mg/kg/day alternate-day dose group, the geometric mean concentration was 7.3 nmol/L at 1 h post-LDD. A similar concentration of DB829 was detected at 2 days post-LDD before decreasing below the limit of quantification.

## Discussion

A variety of novel diamidines and corresponding prodrugs, notably the prodrugs pafuramidine and DB844, have demonstrated high activity against African trypanosomes both *in vitro* and in mouse models of first and second stage HAT [10, 11, 27]. Recently, it was demonstrated that the prodrug DB868 was better tolerated than pafuramidine in rats [28]. Oral DB868 was demonstrated further to be well tolerated and highly active in a vervet monkey model of first stage HAT, supporting DB868 as a potential clinical candidate for first stage human disease [14]. In the current study, the efficacy and pharmacokinetics of oral DB868 and its active diamidine, IM DB829, were investigated in the vervet monkey model of second stage HAT.

IM DB829 at all dose regimens tested cured all monkeys with second stage HAT, whereas oral DB868 cured a combined 2/10 monkeys. These results indicate improved activity of IM DB829 compared to oral DB868 and other previously evaluated oral prodrug analogues, including pafuramidine, which cured 0/3 monkeys [12], and DB844, which cured 3/7 monkeys [13]. The efficacy of IM DB829 also was superior to that of IM pentamidine, which showed a moderate cure rate (2/3) in first stage disease [14] but was ineffective (0/3) when treatment initiation was delayed to 14 days post-infection with *T. b. rhodesiense* KETRI 2537 (unpublished data, TRC-KARI). Another study evaluating the efficacy of diminazene aceturate and other experimental diamidines against second stage *T. b. rhodesiense* infections in monkeys reported a low cure rate (2/8), with a majority of the monkeys relapsing and progressing to meningo-encephalitis [18]. The current results indicate that, unlike other diamidines and diamidineprodrugs previously evaluated in this monkey model, DB829 potentially could be developed as therapy for second stage HAT.

The geometric mean plasma C_max_ of DB829 after IM administration of 5 and 2.5 mg/kg/day was 25 and 10-times greater, respectively, than that after oral administration of DB868 at 20 mg/kg/day. These results could reflect presystemic loss of DB868, intermediate metabolites, and/or DB829 after oral administration of DB868; however, additional studies are needed to confirm this contention. Although the prodrug strategy improves delivery of dicationic drug molecules through biologic membranes [29], making oral administration of these compounds an attractive therapeutic option especially relevant for resource-poor HAT endemic regions [13], our current findings indicate that parenteral administration may be preferred for the second stage cases due to the superior systemic exposure of active compounds. The long half-life for DB829 (33–180 days) given IM or orally as DB868 is consistent with the dicationic nature of DB829. The observation that the AUC and terminal elimination half-life for the alternate-day DB829 regimen were 5-times greater than those for the consecutive-day dosing regimens is not easily explained. More studies are needed to verify, and explain, these results.

Evaluation of CSF from monkeys treated with IM DB829 at 5 mg/kg/day following either dosing regimen suggested that DB829 penetrated the BBB, attaining peak geometric mean concentrations at 1 h post-LDD of approximately 14 nmol/L (consecutive-day dosing) or 7.3 nmol/L (alternate-day dosing). These concentrations were approximately 0.1% of corresponding total (bound + unbound) plasma concentrations at the 1 h post-LDD time point. All the IM treated monkeys were cured, implying that these concentrations attained the minimum threshold required for efficacy. Indeed, these concentrations compared favourably with the *in vitro* IC_50_ for DB829 (14 nmol/L) determined for *T. b. rhodesiense* STIB900 [11]. In addition, DB829 CSF concentrations were maintained above 5 nmol/L for 2–28 days post-LDD. The persistence of DB829 in CSF may have enabled trypanosomes to accumulate potentially lethal drug concentrations as found previously following pharmacological studies of DB844 in monkeys [13]; *in vitro* accumulation and distribution studies of DB75 and DB820 in African trypanosomes led to similar conclusions [30]. The success of DB829 in curing second stage infection, despite seemingly low CSF concentrations, is consistent with melarsoprol and eflornithine, both of which have a historically high success rate against second stage HAT [3] despite attaining comparably low CSF:plasma ratios [31, 32].

Drug concentrations were not quantifiable in CSF of monkeys treated with the generally unsuccessful oral DB868 treatment regimens, suggesting that DB829 either did not penetrate the BBB or that CSF/CNS concentrations attained were insufficient to cure. The relapsed monkeys manifested a more advanced second stage disease compared to primary infections in the same individuals, substantiating the important role of the CNS as a reservoir of drug-evading, relapse-causing trypanosomes [13]. In addition, parasitaemia in the relapsed monkeys did not reach the peak levels observed in primary parasitaemia (Fig. 5) and was more comparable to the pattern observed in *T. b. gambiense* infections in vervet monkeys [33] (unpublished data, TRC-KARI); human *T. b. gambiense* infections, which comprise more than 95% of HAT, are similarly characterised by very low parasitaemias [3, 34, 35]. Our study did not elucidate the factors responsible for the generally lower parasite loads detected in the blood of relapse as compared to primary infections in the same monkeys. However, it has been reported previously that blood parasite loads in *T. brucei. spp* infections are a product of density-dependent parasite differentiation to the non-replicating short stumpy forms, as well as the killing of parasites by the host’s immune system [36]. Our results are suggestive of a predominance of short stumpy forms and/or a more efficient killing of successive trypanosome variant antigenic types by the immune system in the chronic relapse infections in monkeys.

Time to relapse data for monkeys treated with DB868 at 20 mg/kg/day, a median 133 (range, 77–161) days post-LDD, was a positive indicator that the protracted timelines observed before declaration of cure in preclinical studies in the monkey model [12, 13] and in routine management of HAT patients [3] are not without justification. Primary efficacy endpoint assessments at 30 or 100 days post-LDD have been proposed to improve the approval times for compounds in development for second stage HAT. In our study, a primary efficacy endpoint of 30 days post-LDD would have had little or no value in predicting the final efficacy outcome. However, a primary efficacy endpoint of 100 days post-LDD would have predicted correctly the efficacy outcome for the oral DB868 10 mg/kg/day group but not for the 20 mg/kg/day group since 2/3 relapses in this group were diagnosed as relapses after 100 days post-LDD of DB868. Nevertheless, a review of larger data sets of monkeys treated with different experimental diamidines in our laboratory revealed that a primary endpoint at 100 days post-LDD would have facilitated detection of 20/25 (80%) animals that eventually relapsed (unpublished data, TRC-KARI), indicating that 100 days may be a useful endpoint for go-no go decisions during preclinical drug trials.

Haematology changes attributable to trypanosome infection and subsequent treatment with diamidines were generally consistent with previous observations in this model [12, 13, 37]. However, the current study focused more on total and differential WBC counts in blood. Although WBC changes exhibited wide variations, the finding that WBC remained elevated in monkeys that eventually relapsed, suggests that it could be investigated further as a potential surrogate marker for cure assessment in this model. Other potential surrogate tests for cure assessment include WBC counts in CSF [38], serum/plasma trypanosome antigen levels as determined using TrypTECTT CIAT [23], HAT ELISA [39], and a variety of DNA-based polymerase chain reaction (PCR) and loop-mediated isothermal amplification (LAMP) techniques [40, 41]. However, all these surrogate tests require validation to be used as biomarkers of cure/relapse in drug trials. Other significant haematological changes observed in our current study included elevation in WBC counts, especially lymphocytes and granulocytes, during and immediately post-trypanocidal therapy (Fig. 7A and 7B). The WBC changes were most pronounced post-treatment with melarsoprol and likely reflected rapid destruction of trypanosomes, the release of high amounts of trypanosome antigens (antigenaemia) and associated stimulatory effects on the host’s immune system; however, the significance of these WBC changes needs to be verified in a future study. Importantly, the elevated leukocyte numbers returned to baseline within approximately four days post-LDD of curative melarsoprol therapy (Fig. 7B).

In summary, the current study demonstrated that 10-day dose regimens of the oral diamidine prodrug DB868 did not yield satisfactory efficacy in the monkey model of second stage HAT, similar to the oral prodrug analogue DB844 [13]. Further studies are needed to determine whether alternate regimens in which the prodrug is administered at higher daily doses for shorter durations could improve efficacy without compromising safety. A low dose (2.5 mg/kg/day) of the active diamidine DB829, administered IM for 5 consecutive days, was identified as a promising new treatment that could enter the development pipeline for second stage HAT. It has previously been reported that DB829 was effective against multiple *T. b. rhodesiense and T. b. gambiense* isolates in vitro and in murine models of HAT [42, 43], which when taken together with the current results, indicates that DB829 should be considered a potential clinical candidate for both forms of HAT.
